# Visceral fat obesity is the key risk factor for the development of reflux erosive esophagitis in 40–69-years subjects

**DOI:** 10.1007/s10388-021-00859-5

**Published:** 2021-06-12

**Authors:** Shinya Ohashi, Takahisa Maruno, Keita Fukuyama, Osamu Kikuchi, Tomohiko Sunami, Yuki Kondo, Seiichiro Imai, Aki Matsushima, Kazuyo Suzuki, Fumika Usui, Masahiro Yakami, Atsushi Yamada, Hiroyoshi Isoda, Shigemi Matsumoto, Hiroshi Seno, Manabu Muto, Mayumi Inoue

**Affiliations:** 1 Preemptive Medicine and Lifestyle Disease Research Center, Kyoto University Hospital, 53 Kawahara-cho, Shogoin, Sakyo-ku, Kyoto, 606-8397 Japan; 2 Department of Therapeutic Oncology, Graduate School of Medicine, Kyoto University, 54 Kawahara-cho, Shogoin, Sakyo-ku, Kyoto, 606-8507 Japan; 3 Department of Gastroenterology and Hepatology, Graduate School of Medicine, Kyoto University, 54 Kawahara-cho, Shogoin, Sakyo-ku, Kyoto, 606-8507 Japan; 4 Department of Real Word Data Research and Development, Graduate School of Medicine, Kyoto University, 54 Kawahara-cho, Shogoin, Sakyo-ku, Kyoto, 606-8507 Japan

**Keywords:** Reflux erosive esophagitis, Visceral fat obesity, Visceral fat area, Abdominal computed tomography

## Abstract

**Background:**

Visceral fat obesity can be defined quantitatively by abdominal computed tomography, however, the usefulness of measuring visceral fat area to assess the etiology of gastrointestinal reflux disease has not been fully elucidated.

**Methods:**

A total of 433 healthy subjects aged 40–69 years (234 men, 199 women) were included in the study. The relationship between obesity-related factors (total fat area, visceral fat area, subcutaneous fat area, waist circumference, and body mass index) and the incidence of reflux erosive esophagitis was investigated. Lifestyle factors and stomach conditions relevant to the onset of erosive esophagitis were also analyzed.

**Results:**

The prevalence of reflux erosive esophagitis was 27.2% (118/433; 106 men, 12 women). Visceral fat area was higher in subjects with erosive esophagitis than in those without (116.6 cm^2^ vs. 64.9 cm^2^, respectively). The incidence of erosive esophagitis was higher in subjects with visceral fat obesity (visceral fat area ≥ 100 cm^2^) than in those without (61.2% vs. 12.8%, respectively). Visceral fat obesity had the highest odds ratio (OR) among obesity-related factors. Multivariate analysis showed that visceral fat area was associated with the incidence of erosive esophagitis (OR = 2.18), indicating that it is an independent risk factor for erosive esophagitis. In addition, daily alcohol intake (OR = 1.54), gastric atrophy open type (OR = 0.29), and never-smoking history (OR = 0.49) were also independently associated with the development of erosive esophagitis.

**Conclusions:**

Visceral fat obesity is the key risk factor for the development of reflux erosive esophagitis in subjects aged 40–69 years.

**Supplementary Information:**

The online version contains supplementary material available at 10.1007/s10388-021-00859-5.

## Introduction

The prevalence of gastroesophageal reflux disease (GERD) has clearly increased over the past 20 years in Japan [1, 2]. Chronic inflammation because of GERD leads to an increased risk of esophageal adenocarcinoma [3], which is expected to increase in Japan [4]. Therefore, it is desirable to understand further the etiology of GERD and to use this information to establish preventive strategies for GERD and esophageal adenocarcinoma.

The incidence of obesity is also increasing in Japan because of sedentary lifestyles and changes in diet [5]. Obesity is usually associated with an increase in visceral and/or subcutaneous fat [6]. Individuals with visceral fat accumulation are at particularly high risk for common medical complications, including diabetes and cardiovascular disease [7, 8]. Although body mass index (BMI) is used to evaluate obesity [9], BMI does not measure body fat directly [10]. Abdominal computed tomography (CT) scans can measure fat volume directly and quantitatively, thereby allowing the separate analysis of visceral and/or subcutaneous fat volumes [11, 12]. According to the diagnostic criteria of the Japan Society for the Study of Obesity, individuals with visceral fat area (VFA) ≥ 100 cm^2^ are diagnosed as visceral fat obese [5]. This criterion is used for risk assessment of obesity-related disorders [12, 13].

The relationship between obesity and the incidence of GERD has been evaluated in Japanese populations, and a high BMI has been shown to be an important risk factor for GERD [1, 14]. However, the usefulness of VFA measurement in GERD risk assessment has not been fully elucidated. In this study, we evaluated the relationship between visceral fat obesity as defined by abdominal CT and the risk of reflux erosive esophagitis in age-specific (40–69 years) healthy Japanese subjects and further assessed the various factors involved in the development of erosive esophagitis.

## Methods

### Study design

**Fig. 1:**
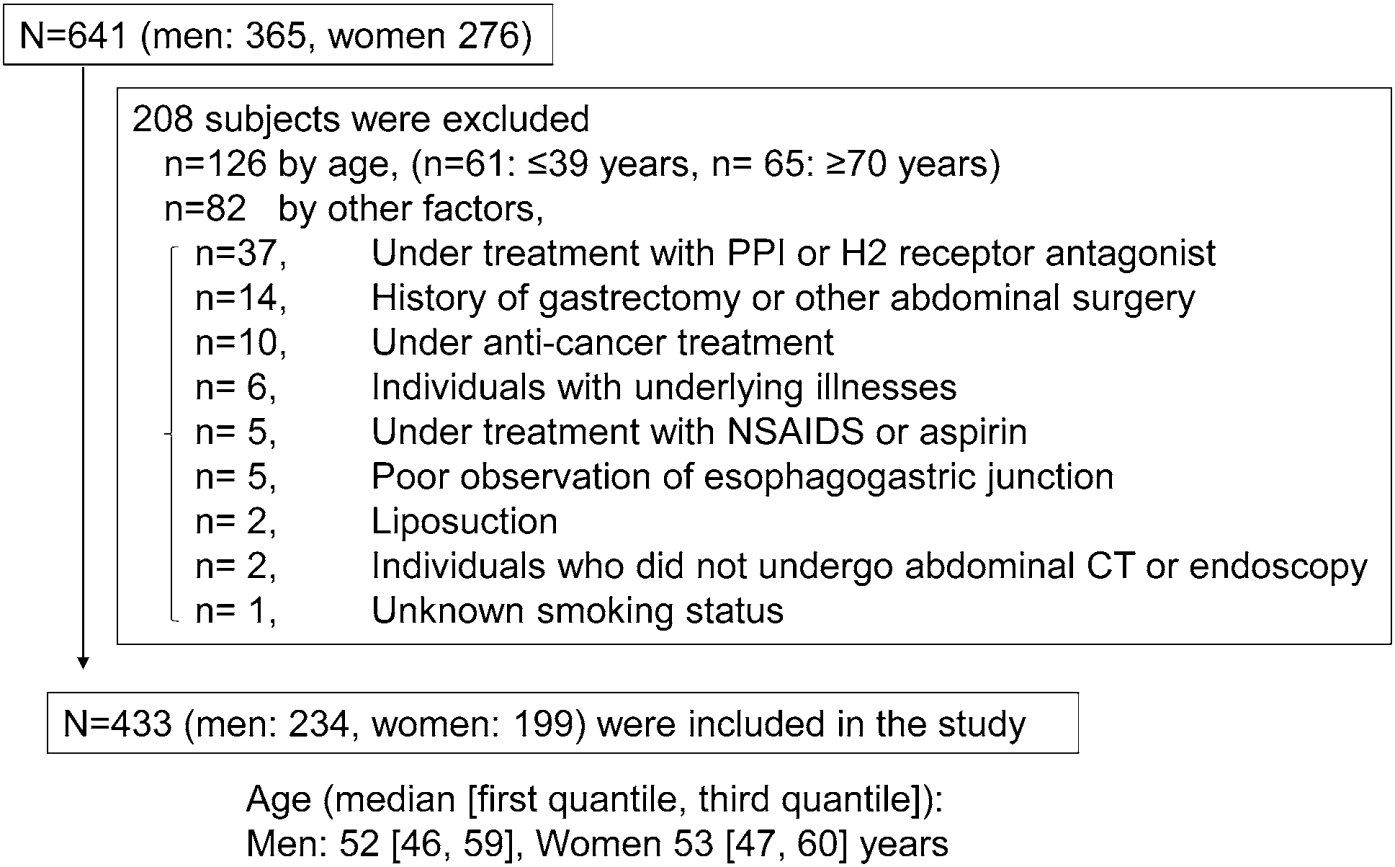
Subject selection. A total of 641 subjects were received in our facility for a medical checkup from 2017.12 to 2018.6, and 433 (men 234, women 199) subjects were included in the analysis (208 subjects were excluded by the criteria as described)

The study subjects were 641 adults who visited the Preemptive Medicine and Lifestyle Disease Research Center in Kyoto University Hospital for a medical checkup. This study targeted healthy adults aged 40–69 years, with reference to the defined target ages for lifestyle-related diseases [15]. To eliminate the effects of drugs that may affect the presence or absence of GERD, individuals being treated with proton pump inhibitors (PPIs) or H2-receptor antagonist (H2-blockers) were excluded. To eliminate subjects who were not healthy, individuals who were receiving anticancer treatment or who had underlying illnesses were excluded. To eliminate the effects of drug-mediated mucosal damage, individuals being treated with nonsteroidal anti-inflammatory drugs (NSAIDs) or aspirin were excluded. In addition, individuals who had a history of gastrectomy or other abdominal surgery, and/or individuals for whom data analysis was not possible, were excluded (Fig. 1). Subjects were asked about the presence or absence of heartburn, smoking Brinkman index (BI), smoking status, alcohol consumption volume, and the presence or absence of flushing reaction by alcohol intake.

This study was carried out in accordance with the Declaration of Helsinki and the protocol was approved by the ethics committee of the Kyoto University Hospital (R0619 and R2576). Written informed consent, which indicated that clinical data without individual information would be used for the study, was obtained from all subjects.

### Upper gastrointestinal endoscopy

Reflux erosive esophagitis was diagnosed by mucosal injuries of grade A or worse according to the Los Angeles classification [16]. Esophageal hiatal hernia was defined as apparent separation of the esophagogastric junction and the diaphragm impression at endoscopy by greater than 2 cm [17]. Gastric mucosal atrophy was assessed according to the Kimura–Takemoto classification [18]. Endoscopic images were reviewed by three board-certified endoscopic specialists.

### Obesity assessment

BMI above normal limits was defined as BMI ≥ 25 kg/m^2^ [5]. Total fat area (TFA), VFA, subcutaneous fat area (SFA), fat area ratio (VFA/SFA) and waist circumference were obtained from cross sectional CT scans at the level of the umbilicus using an image processing workstation (Ziostation2; Ziosoft Corp, Tokyo, Japan). Individuals with VFA ≥ 100 cm^2^ were defined as visceral fat obese [5].

### Categorization of obesity by VFA and BMI

In this study, we used the following four categories to classify obesity in subjects: category A. BMI < 25 kg/m^2^ and VFA < 100 cm^2^; category B. BMI < 25 kg/m^2^ and VFA ≥ 100 cm^2^; category C. BMI ≥ 25 kg/m^2^ and VFA < 100 cm^2^; and category D. BMI ≥ 25 kg/m^2^ and VFA ≥ 100 cm^2^.

### Statistical analyses

Factors associated with the presence or absence of erosive esophagitis were evaluated. Normality of distribution was assessed by Shapiro–Wilk test. The continuous variables that were not normally distributed were expressed as median [first quantile, third quantile]. Univariable analyses were conducted to assess the difference in risk factors between subjects with or without erosive esophagitis. Mann**–**Whitney *U* test or Fisher’s exact test was used to compare continuous or categorical variables, respectively. The influences of obesity-related factors on the development of erosive esophagitis were estimated by calculating the odds ratios (ORs) and 95% confidence intervals using logistic regression analysis. For the multivariate analysis, we selected variables based on prior knowledge [19–21] according to the previous key reports on the pathophysiology and epidemiology of GERD [1, 22–25]. Hence, the following eight variables, i.e., sex, obesity (visceral fat obesity and subcutaneous fat obesity), presence of esophageal hiatal sliding hernia, stomach conditions (gastric atrophy open type, and *Helicobacter pylori* [*H. pylori*] antibody positivity), and lifestyle factors (average daily alcohol intake and smoking history) were subjected to multivariate analysis. All statistical analyses and visualizations were performed using Microsoft Excel and R (version 3.6.3) with Rstudio (1.2.5033). R packages, ggVennDiagrama (0.3), officer (0.3.11), cowplot (1.0.0), sf (0.9–6), lawstat (3.4), ggpubr (0.3.0), MASS (7.3–51.5), and tidyverse (1.3.0). Differences at *P* < 0.05 were considered statistically significant.

## Results

### Subjects

A total of 208 subjects met the exclusion criteria and were excluded, leaving a total of 433 subjects (234 men and 199 women) for the analysis (Fig. 1). The median ages for men and women were 52 and 53 years, respectively.

### Basic characteristics of obesity-related factors by gender

The median BMI, abdominal diameter, TFA, VFA, and fat area ratio values were significantly higher in men than in women, although there was no difference in the median SFA value. The average serum adiponectin level was significantly lower in men than in women (Table 1).

**Table 1:** Characteristics of participants on obesity indexes by gender

Obesity indexes	Men	Women	*P* value
Body mass index (BMI, kg/m^2^)	24.6 [23.1, 26.7]	20.9 [19.2, 23.5]	< 0.001^†^
Waist circumference (cm)	87.4 [82.6, 92.8]	78.5 [72.7, 84.2]	< 0.001^†^
Total fat area (TFA, cm^2^)	238.3 [195.6, 298.2]	195.7 [135.6, 267.2]	< 0.001^†^
Visceral fat area (VFA, cm^2^)	97.3 [68.9, 126.8]	45.5 [26.0, 76.6]	< 0.01^†^
Subcutaneous fat area (SFA, cm^2^)	138.4 [108.4, 177.3]	149.3 [100.3, 192.9]	0.47^†^
Fat area ratio (VFA/SFA)	0.70 [0.47, 0.90]	0.32 [0.24, 0.43]	< 0.001^†^
Serum adiponectin (μg/mL)	6.4 [4.8, 8.4]	12.2 [8.4, 17.1]	< 0.001^†^

Data are expressed as the median [first quantile, third quantile]

^†^Mann**–**Whitney *U* test

### Diagnosis of erosive esophagitis

Overall, 118 subjects (106 men, 12 women) were diagnosed with erosive esophagitis, so the prevalence of erosive esophagitis was 27.3% (118/433, Grade A: 111, B: 7, C: 0, D: 0), and all cases of esophagitis in enrolled subjects were mild. The intra-observer concordance rate (*κ* value) was 82.8%. The prevalence of erosive esophagitis in men was 45.3% (106/234), whereas that in women was 6.0% (12/199) (Table 2). The prevalence of erosive esophagitis in subjects with heartburn symptoms was significantly higher than in those without (49.0% [25/51] vs. 24.3% [93/282], respectively); 22% of subjects (25/118) with erosive esophagitis had heartburn symptoms.

**Table 2:** Comparison of clinical characteristics between subjects with or without erosive esophagitis

	Non-erosive esophagitis (*n* = 315)	Erosive esophagitis (*n* = 118)	*P* value	Percentage
Age (year)^a^	52.0 [46.5, 60.0]	52.0 [46.0, 59.0]	0.62^†^	
Sex			< 0.001^‡^	
Men	128	106		45.3% (106/234)
Women	187	12		6.0% (12/199)
Heartburn			< 0.001^‡^	
(−)	289	93		24.3% (93/382)
(+)	26	25		49.0% (25/51)
Adiponectin (μg/mL)^a^	9.3 [6.4, 14.1]	5.8 [4.32, 8.40]	< 0.001^†^	
Visceral fat area cut-off 100 cm^2^			< 0.001^‡^	
Non-visceral fat obesity (< 100 cm^2^)	265	39		12.8% (39/304)
Visceral fat obesity (≥ 100 cm^2^)	50	79		61.2% (79/129)
BMI cut-off 25			< 0.001^‡^	
Non-obesity (BMI < 25)	250	55		18.0% (55/305)
Obesity (BMI ≥ 25)	65	63		49.2% (63/128)
Smoking Brinkman index (BI)^a^	0.0 [0.0, 390.0]	405.0 [100.0, 678.8]	< 0.001^†^	
Smoking status			< 0.001^‡^	
Current smoker	52	33		38.8% (33/85)
Ex-smoker	107	63		37.1% (63/170)
Never smoker	156	22		12.3% (22/178)
Daily alcohol intake (g/day)^a^	10.0 [0.0, 40.0]	44.0 [15.0, 70.0]	< 0.001^†^	
Flushing reaction by alcohol intake			0.549^‡^	
Flushing reaction negative	179	65		26.6% (65/244)
Flushing reaction positive	73	24		24.7% (24/97)
Flushing reaction unknown	63	29		31.5% (29/92)
Anti-*H. pylori* antibody			0.035^‡^	
(–)	251	105		29.5% (105/356)
(+)	64	13		16.8% (13/77)
Gastric atrophy			0.162^‡^	
Closed type	252	102		28.8% (102/354)
Open type	63	16		20.2% (16/79)
Pepsinogen I^a^	47.4 [38.8, 58.2]	51.8 [41.8, 63.3]	0.04^†^	
Pepsinogen II^a^	8.4 [6.7, 10.1]	8.4 [6.9, 10.3]	0.85^†^	
Pepsinogen I/II ratio^a^	5.8 [4.9, 6.6]	6.2 [5.2, 7.0]	0.005^†^	
Pepsinogen I/II ratio cut-off 3			0.02^‡^	
≥ 3	296	117		28.3% (117/413)
< 3 (= severe atrophy)	19	1		5.0% (1/20)
Esophageal hiatal hernia			< 0.001^‡^	
(−)	258	35		11.9% (35/293)
(+)	57	83		59.2% (83/140)

^a^Data for continuous variables are expressed as the median [first quantile, third quantile]

^†^Mann**–**Whitney *U* test

^‡^Fisher’s exact test

### Comparison of clinical examinations between patients with or without erosive esophagitis

**Fig. 2:**
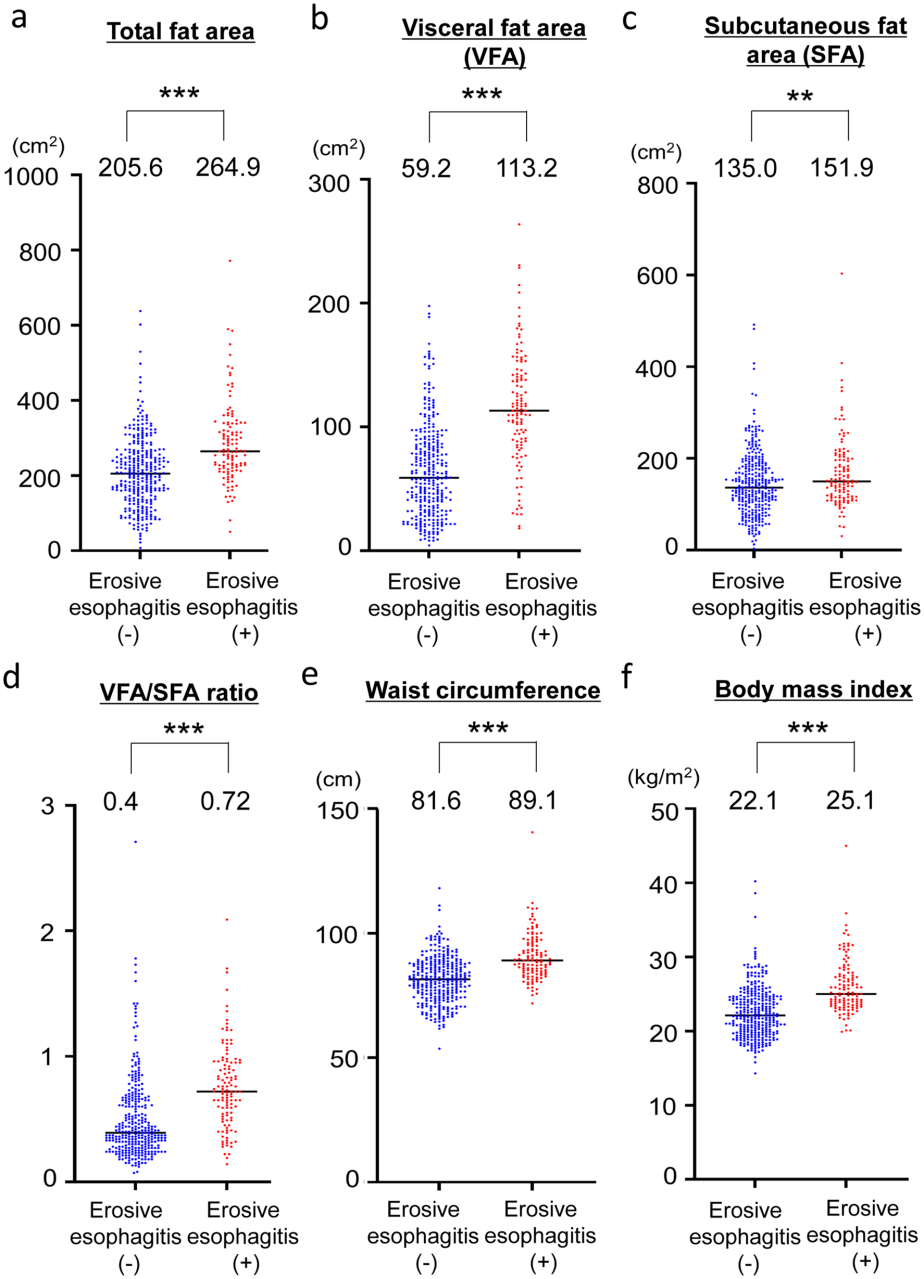
Comparison of obesity-related factors between subjects with or without erosive esophagitis. **a** Total fat area, **b** visceral fat area (VFA), **c** subcutaneous fat area (SFA), **d** VFA/SFA ratio, **e** waist circumference determined by abdominal CT, and **f** body mass index (BMI). The median value in each group is indicated. ***P < 0.001, **P < 0.01

All obesity-related factors (BMI, TFA, VFA, SFA, fat area ratio, and waist circumference) were significantly higher in subjects with erosive esophagitis than in those without (Fig. 2). As shown in Table 2, serum adiponectin level was significantly lower in subjects with erosive esophagitis than in those without.

Regarding the relationship between visceral fat obesity and the incidence of erosive esophagitis, 61.2% (79/129) of subjects with visceral fat obesity (VFA ≥ 100 cm^2^) had erosive esophagitis, and 66.9% (79/118) of subjects with erosive esophagitis were visceral fat obese. Among the subjects without visceral fat obesity, 87% (265/304) did not have erosive esophagitis.

As for BMI, 49.2% (63/128) of subjects with high BMI (≥ 25 kg/m^2^) had erosive esophagitis, and 53.3% (63/118) of subjects with erosive esophagitis had a high BMI; 82% (250/305) of subjects with normal BMI (< 25 kg/m^2^) did not have erosive esophagitis.

Cumulative smoking BI values were significantly higher in subjects with erosive esophagitis than in those without. The prevalence of erosive esophagitis in current smokers, ex-smokers, and never-smokers was 38.8%, 37.1%, and 18.6%, respectively.

Current daily alcohol consumption was significantly higher in subjects with erosive esophagitis than in those without (44.0 vs.10.0 g/day, respectively). The presence or absence of a flushing reaction did not affect the prevalence of erosive esophagitis.

Regarding the relationship between the presence or absence of *H. pylori* antibodies and the incidence of erosive esophagitis, the prevalence of erosive esophagitis was significantly higher in those who were negative for *H. pylori* antibodies (29.5%) than in those who were positive for *H. pylori* antibodies (16.8%).

Regarding the relationship between gastric atrophy and the incidence of erosive esophagitis, the prevalence of erosive esophagitis in subjects who were classified into each grade of gastric mucosal atrophy into each category is shown in Online Resource 1. The prevalence of erosive esophagitis in subjects diagnosed with closed type atrophy or open type atrophy was 28.8% (102/354) and 20.2% (16/79), respectively. The prevalence of erosive esophagitis in subjects with a pepsinogen I/II ratio ≥ 3 or < 3, which is the cutoff value to determine severe gastric atrophy [26], was 28.3% and 5.0%, respectively.

In addition, the prevalence of erosive esophagitis in subjects with esophageal sliding hernia was 59.2%, whereas that in subjects without esophageal sliding hernia was 11.9%. VFA was significantly higher in subjects with esophageal hernia than in those without (118.1 cm^2^ vs. 60.2 cm^2^, respectively, Online Resource 2).

### Univariate analysis

Univariate analysis with continuous variables revealed that BMI, waist circumference, TFA, VFA, SFA, fat area ratio, serum adiponectin level, daily alcohol consumption level, cumulative smoking BI, and pepsinogen I/II ratio were significantly associated with the risk of erosive esophagitis (Table 3).

**Table 3:** Effect of obesity on erosive esophagitis by univariate (continuous and/or non-continuous variables) and multivariate analysis

Category (continuous variables)	Adjusted OR (95% CI)	*P* value	Adjusted coefficient
Univariate analysis			
Age	0.94 (0.76–1.17)	0.62	− 0.057
Body mass index (BMI)	2.83 (2.15–3.73)	< 0.01	1.04
Waist circumference	3.02 (2.26–4.03)	< 0.01	1.11
Total fat area (TFA)	2.25 (1.75–2.89)	< 0.01	0.81
Visceral fat area (VFA)	3.73 (2.78–4.99)	< 0.01	1.32
Subcutaneous fat area (SFA)	1.41 (1.14–1.74)	< 0.01	0.34
Fat area ratio (VFA/SFA)	2.04 (1.62–2.58)	< 0.01	0.71
Adiponectin	0.29 (0.2–0.43)	< 0.01	− 1.23
Daily alcohol intake (g/day)	1.83 (1.48–2.27)	< 0.01	0.61
Smoking Brinkman Index	1.94 (1.56–2.42)	< 0.01	0.66
Pepsinogen I/II ratio	1.41 (1.13–1.76)	< 0.01	0.34

Category (non-continuous variables)	OR (95% CI)	*P* value	Coefficient
Univariate analysis			
Gender male	12.9 (6.82–24.42)	< 0.01	2.56
Visceral fat area ≥ 100 cm^2^	10.74 (6.59–17.5)	< 0.01	2.37
BMI ≥ 25 kg/m^2^	4.41 (2.8–6.93)	< 0.01	1.48
Heartburn positive	2.99 (1.65–5.43)	< 0.01	1.09
Esophageal hiatal hernia positive	10.73 (6.59–17.49)	< 0.01	2.37
Never-smoking history	0.23 (0.14–0.39)	< 0.01	− 1.45
Anti-*H. pylori* antibody positive	0.49 (0.26–0.92)	0.024	− 0.72
Gastric atrophy open type	0.63 (0.35–1.14)	0.12	− 0.47
Flushing reaction positive	0.91 (0.53–1.56)	0.32	− 0.099

Category	OR (95% CI)	*P* value	Coefficient
Multivariate analysis			
Gender male	3.36 (1.47–7.67)	< 0.01	1.21
Visceral fat area	2.18 (1.45–3.26)	< 0.01	0.78
Subcutaneous fat area	1.26 (0.89–1.78)	0.20	0.23
Daily alcohol intake (g/day)	1.54 (1.15–2.06)	< 0.01	0.43
Gastric atrophy open type	0.29 (0.13–0.66)	< 0.01	− 1.24
Anti-*H. pylori* antibody positive	0.44 (0.19–1.01)	0.053	− 0.83
Never-smoking history	0.49 (0.25–0.96)	0.039	− 0.72
Esophageal hiatal hernia positive	3.73 (1.99–6.99)	< 0.01	1.31

Univariate analysis with non-continuous variables revealed that male gender, VFA ≥ 100 cm^2^, BMI ≥ 25 kg/m^2^, heartburn positive, esophageal hiatus hernia positivity, never-smoking history, and *H. pylori* antibody positivity were associated with the risk of erosive esophagitis (Table 3).

### Multivariate analysis

Male gender (OR 3.36), VFA (OR 2.18), daily alcohol intake (OR 1.54), and esophageal hiatal hernia (OR 3.73) were independent risk factors for erosive esophagitis. Gastric atrophy open type (OR 0.29) and never-smoking history (OR 0.49) were independent negative risk factors for erosive esophagitis (Table 3). The area under the curve (AUC) of the receiver-operating characteristics (ROC) curve calculated from the multivariate analysis was 0.895 (Online Resource 3), and the data for the estimated predictive probability are shown in Online Resource 4.

### Distribution of visceral fat area by gender in subjects with or without erosive esophagitis

**Fig. 3:**
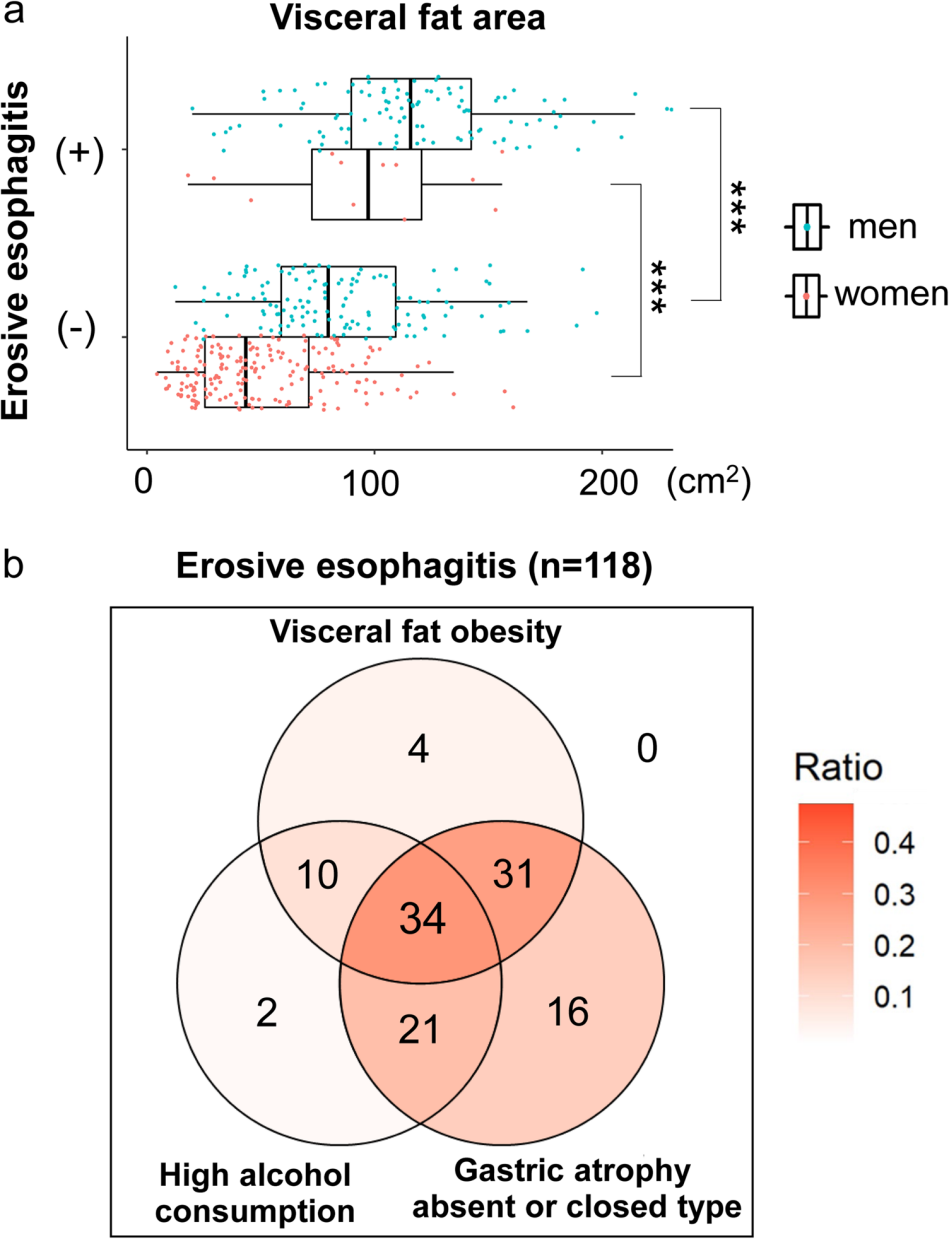
Distribution of subjects with or without erosive esophagitis based on visceral fat area by sex, and of subjects with erosive esophagitis based on three factors: visceral fat obesity, high alcohol consumption (≥ 30 g/day), and gastric atrophy absent or closed type. **a** Box plot showing the distribution of subjects with or without erosive esophagitis on visceral fat area in men and women. ***P < 0.001 between groups. **b** Venn diagram showing the relationship between the three factors and subjects with erosive esophagitis. The number of subjects applicable to each section is indicated. Color code is shown according to the ratio

The median VFA in subjects with erosive esophagitis was significantly higher than that in those without, for both men (115.9 cm^2^ vs.79.6 cm^2^, respectively) and women (97.3 cm^2^ vs.43.5 cm^2^, respectively, Fig. 3a).

### Distribution of subjects with erosive esophagitis regarding visceral fat obesity, high alcohol consumption, and gastric atrophy

Regarding the etiology of reflux erosive esophagitis, we focused particularly on visceral fat obesity, high-level alcohol drinkers, and gastric atrophy (absent or closed type) based on endoscopic findings. As shown, 34 (28.8%) of 118 subjects with erosive esophagitis had all three factors, and 102 subjects (86.4%: 102/118) were either visceral fat obese or drinking ≥ 30 g alcohol/day (Fig. 3b).

### Distribution of subjects with erosive esophagitis classified by visceral fat area and BMI

**Fig. 4:**
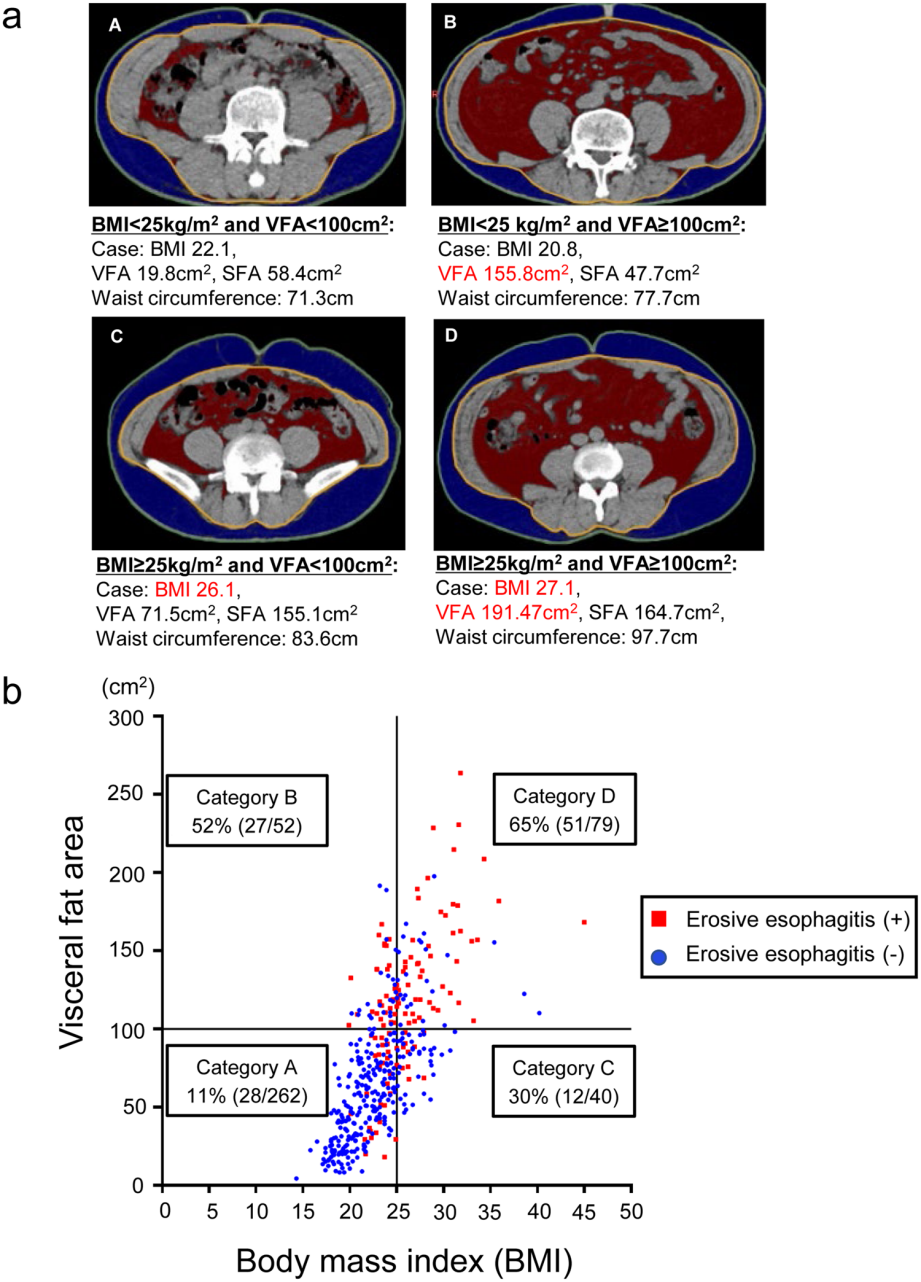
Representative abdominal CT images of four subjects according to body fat composition and the distribution of participants in each category. **a** Abdominal CT images of four cases representing four categories (A, B, C, and D) classified by visceral fat area and BMI. (A) BMI < 25, visceral fat area (VFA) < 100 cm^2^; (B) BMI < 25, VFA ≥ 100 cm^2^; (C) BMI ≥ 25, VFA < 100 cm^2^; (D) BMI ≥ 25, VFA ≥ 100 cm^2^. **b** Distribution of subjects with erosive esophagitis in four categories. Positive rate of subjects with erosive esophagitis in each category is shown: (A) 11% [28/262]; (B) 52% [27/52]; (C) 30% [12/40]; and (D) 65% [51/79]

As shown in Fig. 4, the incidence of erosive esophagitis in each category (A, B, C, D) was 11%, 52%, 30%, and 65%, respectively.

## Discussion

The increase in the obese population has coincided with a rising prevalence of GERD [22]. Obesity has been shown to be associated with excessive transient lower esophageal sphincter relaxation, which is considered to play a key role in the pathophysiology of the development of GERD because of the increase in intra-gastric pressure [27]. El-Serag, et al. reported that abdominal obesity was correlated with the increase in intragastric pressure, and noted the possible involvement of visceral fat obesity as a mechanism of obesity-mediated intra-gastric pressure elevation [28].

In this study, we demonstrated that visceral fat accumulation was an independent risk factor for the development of erosive esophagitis in subjects aged 40–69 years. Our results are consistent with those of a previous cohort study [23] and case–control studies [24, 29] conducted in South Korea, as well as a cohort study conducted in Japan [25]. Compared with those studies, this study has the following original point: the subjects analyzed in this study were of a specific age group (40–69 years) in which the prevalence of lifestyle-related diseases increases. In addition, we excluded subjects who were taking medications (e.g., PPI/H2-blocker, NSAIDS/aspirin, and anticancer treatment). We believe that the relationship between visceral fat obesity and reflux erosive esophagitis can be accurately evaluated by excluding other factors that may influence the development of erosive esophagitis. Another original point is that we categorized obesity using VFA and BMI, and analyzed the incidence of erosive esophagitis in each category.

In this study, visceral fat obesity had the highest OR among obesity-related factors. In addition, our original categorization regarding obesity demonstrated a high incidence of erosive esophagitis in Categories B (VFA ≥ 100 cm^2^, and BMI < 25 kg/m^2^) and D (VFA ≥ 100 cm^2^, and BMI ≥ 25 kg/m^2^). It is an important finding that individuals in Category B, which is not identified as a high-risk group by traditional obesity criteria (BMI ≥ 25 kg/m^2^), are also at high risk of reflux erosive esophagitis. The mechanism by which visceral fat obesity increases the incidence of reflux esophagitis most likely involves an increase in intra-abdominal pressure, because this increases esophageal acid exposure [30]. In addition, our results support the concept that visceral fat obesity might be involved in the development of esophageal hernia and the subsequent onset of reflux erosive esophagitis.

Further, we showed that serum adiponectin levels were significantly lower in subjects with erosive esophagitis than in those without, which is consistent with a previous report [31]. The circulating adiponectin level is inversely related to visceral fat accumulation [32]. Accordingly, this result supports our finding that visceral fat obesity is associated with the development of reflux erosive esophagitis.

As for gastric condition, we showed that gastric atrophy open type was an independent negative risk factor for the development of erosive esophagitis. Our data also showed that the incidence of erosive esophagitis in subjects with a pepsinogen I/II ratio < 3.0, which indicates severe atrophy [26], was low compared with that in subjects with a pepsinogen I/II ratio ≥ 3.0. This finding supports the idea that gastric atrophy is a negative risk factor for the development of erosive esophagitis.

In this study, we focused on the following three factors, visceral fat obesity, high alcohol intake (≥ 30 g/day), and gastric atrophy absence or closed type, as the key for developing reflux erosive esophagitis, because visceral fat obesity and daily alcohol intake were independent positive risk factors and gastric atrophy open type was a negative risk factor for erosive esophagitis. Of note, the proportion of subjects with at least one factor was 100% (118/118). We speculate that these three factors are key to understanding the pathophysiology of reflux erosive esophagitis in this age group (40–69 years).

In this study, the AUC of the ROC calculated from the multivariate analysis of subjects 40–69 years of age was 0.895 (Online Resource 3). When we evaluated whether the factors that were identified as independent risk factors for the analysis could be applied to younger (≤ 39 years of age [*n* = 52]) and older populations (≥ 70 years of age [*n* = 40]), the AUC of the ROC was 0.892 and 0.879, respectively (data not shown). Thus, the predictive performance of these risk factors for both the younger (≤ 39 years) and the older (≥ 70 years) groups was similar to that for the group aged 40–69 years. However, we speculate that the predictive performance may decline in patients with underlying illness and/or older patients with osteoporosis and kyphosis. Nevertheless, our analysis may contribute greatly to the prediction of the onset of erosive esophagitis in healthy subjects aged 40–69 years, which is the predominant age group for lifestyle-related diseases.

One limitation of the study is that only healthy subjects were enrolled. Therefore, there may be differences in the etiology of GERD between patients and healthy subjects. In addition, the characteristics and the incidence of GERD in our subjects may differ from those in the general population, because this was a single-center study. Issues such as the low incidence of erosive esophagitis in women and the possibility of a selection bias of enrolled healthy populations are also limitations of this study. Further, the relationship between GERD risk and the changes over time in individual visceral fat volume is unclear because this was a cross-sectional study. In addition, the relationship of visceral fat obesity to the severity of erosive esophagitis cannot be evaluated, because all subjects with erosive esophagitis in this study had mild disease. Therefore, visceral fat obesity is an independent risk factor for mild erosive esophagitis, but it remains to be confirmed whether it is associated with the development of severe erosive esophagitis.

In conclusion, we demonstrated that visceral fat obesity is the key independent risk factor for the development of reflux erosive esophagitis. Our findings provide new insights into the etiology of reflux esophagitis and contribute to the establishment of preventive strategies for reflux erosive esophagitis in subjects aged 40–69 years.

## Supplementary Information

Below is the link to the electronic supplementary material.

Supplementary file1 (PDF 544 KB)
