# Supplementary material for: Visceral fat obesity is the key risk factor for the development of reflux erosive esophagitis in 40–69-years subjects
Source: Esophagus. 2021 Jun 12;18(4):889–99. doi: 10.1007/s10388-021-00859-5 (PMC8387261; doi:10.1007/s10388-021-00859-5)
Supplement: Supplementary file 1 — Supplementary file1 (PDF 544 KB) [file 10388_2021_859_MOESM1_ESM.pdf]

	Non-erosive esophagitis (*n* = 315)	Erosive esophagitis (n = 118)
・Atrophy absent	184	84
・Atrophy grade C-1	10	2
・Atrophy grade C-2	46	9
・Atrophy grade C-3	12	7
・Atrophy grade O-1	37	12
・Atrophy grade O-2	17	3
・Atrophy grade O-3	9	1

Online Resource 1. Number of subjects with or without erosive esophagitis in each category of gastric mucosal atrophy

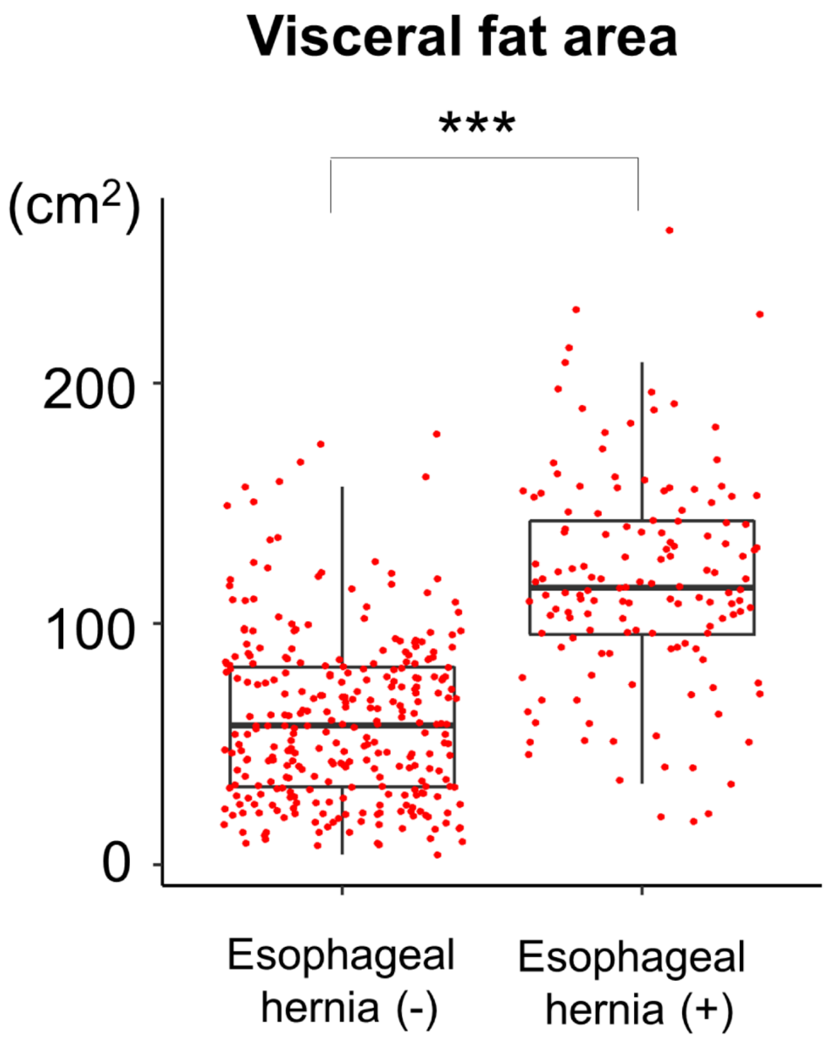

Online Resource 2

Online Resource 2. Comparison of visceral fat area in subjects with or without esophageal hernia. ***$P < 0.001$ between groups.

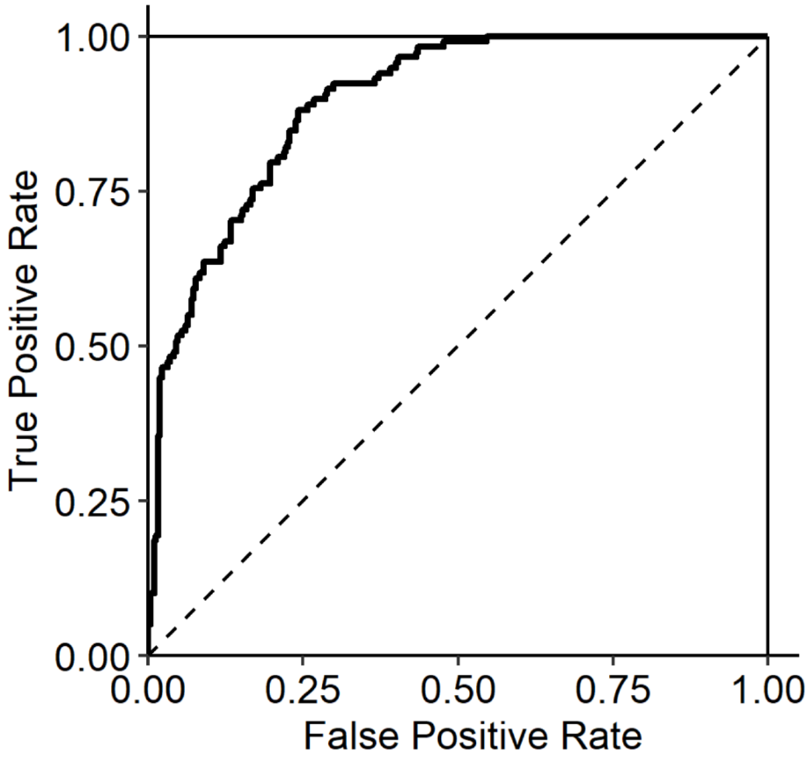

Online Resource 3

Online Resource 3. Receiver operating characteristics curve calculated from the multivariate analysis of subjects 40－69 years of age. The area under the curve was 0.895.

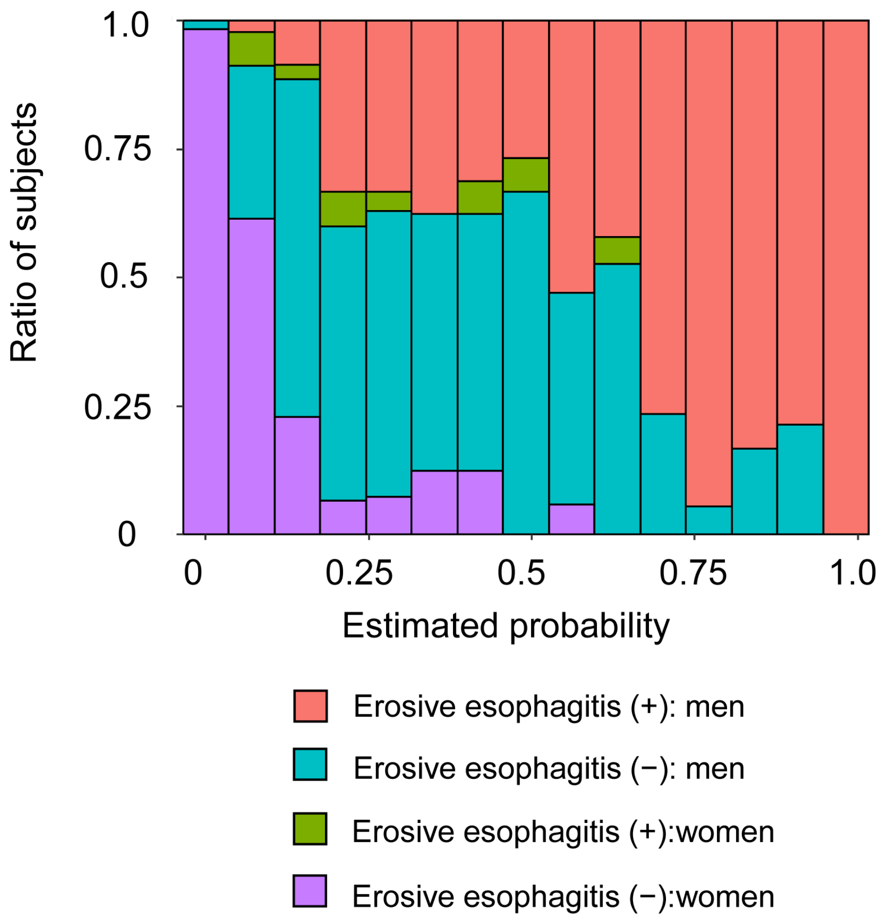

Online Resource 4

Online Resource 4. Estimated predictive probability based on erosive esophagitis and sex calculated from the multivariate analysis.
